# Visceral fat area measured by electrical bioimpedance as an aggravating factor of COVID-19: a study on body composition

**DOI:** 10.1186/s12879-023-08833-5

**Published:** 2023-11-24

**Authors:** Juliana Bonfleur Carvalho, Grasiela Konkolisc Pina de Andrade, Ludiane Alves do Nascimento, Natalia Golin, Ana Lucia Chalhoub Chediac Rodrigues, Erika Suiter, Maryana Virginia Orellana Soprani, Ariane Severine Nadolskis

**Affiliations:** Department of Nutrition, Hospital Sírio Libanês, 91, Dona Adma Jafet, Street, São Paulo, 01308-901 SP Brazil

**Keywords:** Obesity, COVID-19, Bioimpedance electrical, Visceral fat area, Muscle strength

## Abstract

**Introduction:**

Severe forms of COVID-19 are more common in patients with abnormal fat distribution, particularly high visceral adiposity. The patient’s muscle strength may be reduced during the acute phase of the infection. Electrical bioimpedance (BIA) is a non-invasive method for measuring body compartments and estimating visceral fat area (VFA) that can be used at the bedside.

**Objective:**

To assess the association between several body composition parameters, primarily high adipose tissue and high VFA, in patients with and without a diagnosis of COVID-19 infection, and whether it worsened the severity parameters.

**Methods:**

This retrospective cohort study was conducted in a private hospital in the city of São Paulo from March 2020 to August 2021. The demographic and clinical data was collected from medical reports. Body composition is assessed using the InBODY® model S10 bioelectrical impedance device and a Jamar® digital hydraulic manual dynamometer with a scale from 0 to 90 kg is used to measure handgrip strength (HGS).

**Results:**

A total of 96 patients with a mean age of 69.1 years (SD 15) were divided into two groups of 48 individuals, with and without COVID-19 infection. Body mass index (odds ratio [OR]: 4.47, 95% confidence interval [CI]: 1.69, 11.83), fat mass (OR: 2.03, 95% CI: 0.48, 8.55), and VFA (OR: 1.08, 95% CI: 0.33, 3.53) were all higher in the infection group. When COVID-19 patients were evaluated, those with higher VFA had longer hospital stays (OR: 0.99, 95% CI: 0.97, 1.01) and used more vasoactive drugs (*p* = 0.043). Patients with COVID-19 with poor handgrip strength were 3.29 times more likely to require a prolonged intensive care unit (ICU) stay.

**Conclusion:**

The study concluded that excess weight and body fat are significantly associated with COVID-19 involvement, but the severity is primarily related to a greater area of visceral fat. The use of bioimpedance for visceral fat measurement was effective, as it is a simple method performed in the hospital setting that does not require the use of radiation.

## Introduction

Patients with severe COVID-19 usually require prolonged mechanical ventilation and have a case fatality rate of up to 49% [1, 2]. In obese people, the inflammation and weakened immune systems can contribute to developing viral diseases. Do to this obesity among COVID-19 hospitalized patients can reach 61.3% [3] and has a critical impact on both hospital prognosis and post-COVID-19 vulnerability [4].

Severe forms of COVID-19 are also more common in patients with abnormal fat distribution, particularly high visceral adiposity [5, 6]. The content of adipose tissue and.

its distribution can predict the prognosis of COVID-19 and has a significant impact on the immune system [7, 8]. Excess visceral adipose tissue (VAT), which is highly metabolically active, is harmful to several aspects of health. VAT secretes inflammatory mediators that can amplify the cytokine storm triggered by SARS-CoV-2, possibly contributing to the severity of the disease [8].

Furthermore, abdominal obesity is associated with other diseases, such as dyslipidemia, diabetes mellitus, atherosclerosis, and hypertension. During the COVID-19 pandemic, preventive procedures against the spread of COVID-19 have accelerated weight gain and given a negative influence on cardiometabolic profiles [9].

Electrical bioimpedance (BIA) is a non-invasive and validated method for assessing body composition. It is measured by an electric current that passes through the body compartments, which provides resistance and causes a delay in conduction through the membranes, causing reactance [2]. BIA helps estimate the most important body compartments, such as fat mass, fat-free mass, and body water [10]. Also analyses the phase angle (PA) and visceral fat by measuring the visceral fat area (VFA).

As a biomarker for malnutrition and inflammatory diseases, PA has been proposed as a prognostic indicator in clinical practice. It is a unique predictor of mortality in several clinical conditions and an option for practical evaluation and prognosis in hospitalized COVID-19 patients [11–13].

VFA is mostly analyzed using tomography images, but can also be measured using bioimpedance. The main advantages of using bioimpedance are that it may be performed at the bedside and it does not require radiation. VFA is measured in square centimeters by BIA, and its ideal value is less than 100 cm^2^ [14].

This study shows the characteristics of COVID-19 patients and the importance of understanding how the body composition profile can influence the onset and severity of the disease.

## Objective

To investigate the association between body composition parameters, particulary the high total adipose tissue and high visceral fat area in patients with or without COVID-19 infection, and whether it worsened the severity parameters.

## Methods

### Study design

This retrospective cohort study was conducted in a private hospital in the city of São Paulo from March 2020 to August 2021.

Patients were divided into two groups (with and without COVID-19). To calculate the sample, we based ourselves on the article “Nutritional assessment and management of critically ill patients with COVID-19 during post-intensive rehabilitation” [15]. We estimated the difference between two means with independent groups using the median weight of patients before (87.0 kg) and after (75.8 kg) admission to the Critical Unit, with an alpha of 5% and a beta of 20%, and a sample of 40 patients in each group (with COVID-19 and without COVID-19).

COVID-19 patients 18 years old or older who had been admitted to Intensive Care Units met the inclusion criteria. Only patients diagnosed with COVID-19 via polymerase chain reaction in the upper respiratory tract were included in the group of patients affected by COVID-19. All patients whose current reason for hospitalization was not COVID-19 infection were included in the other group (without COVID-19). Patients who lacked information on the control variables and those with contraindications to performing bioelectrical impedance, such as pacemakers and extensive metallic prostheses (e.g., hip and femur), were excluded.

### Search procedures

The following information was collected from medical reports: demographic data such as sex and age; clinical data such as admission diagnosis, length of stay, prolonged hospitalization (> 30 days), length of stay in the intensive care unit (ICU), prolonged ICU stay (> 15 days), need for orotracheal intubation (OTI), use of vasoactive drugs, and nutritional data such as weight, height, and body mass index (BMI) calculated using the equation (weight/height^2^) and ranked according to World Health Organization (WHO) [16] criteria for adults and Pan American Health Organization (PAHO) [17] criteria for the older adults; and body composition data.

The following were considered as severity parameters: using vasoactive drugs, in light of the important contribution of endothelial dysfunction to COVID-19 and its sequelae [18]; prolonged hospital stay and prolonged ICU stay because prolonged bed rest and hospital stay (> 2 weeks) are associated with worse clinical course and long-term sequelae in critical care and this can be extrapolated to COVID-19 [8].

Body composition is assessed as part of an institutional protocol using the InBODY® model S10 bioelectrical impedance device, following all manufacturer recommendations to ensure the highest measurement accuracy.

Body composition data includes the Skeletal Muscle Mass Index, with muscle mass considered low when it is < 7.0 kg/m^2^ for men and 5.7 < kg/m^2^ for women [19]. Fat Mass (FM) in kilograms (Kg), which is considered high when it is 160% above the ideal fat mass for the patient, and VFA, which is considered high when values exceed 100 cm^2^ [14].

A Jamar® digital hydraulic manual dynamometer with a scale from 0 to 90 kg is used to measure handgrip strength (HGS), which is performed in the dominant hand, with the patient sitting, elbow flexed at 90°, forearm and wrist in neutral rotation. When sitting was impossible, the patient was measured while lying down with the elbow flexed at 30° [20]. The largest measure among three measurements was selected, and handgrip strength was considered low when it was < 27 kg for men and < 16 kg for women [21].

### Ethical aspects

The study was submitted to the Research Ethics Committee (REC) of the institution and approved by the committee under the number CAAE: 54571221.2.0000.5461.

The waiver of the Informed Consent Form was granted by the REC because the study is retrospective and uses secondary data. The confidentiality of the research subjects was ensured, and the data gathered will be used solely and exclusively for the execution of this study.

### Statistical analysis

Simple and crossed tables were used for the qualitative or categorized variables in the descriptive data analysis. A mean and standard deviation were used to represent quantitative variables’ central tendency and dispersion. The Shapiro-Wilk test was used to confirm normality so a parametric or non-parametric test could be chosen. The Student’s T test was used to compare the results of quantitative variables in the groups with and without COVID-19. The non-parametric Mann-Whitney test was used where normality could not be established. When dealing with categorical data, Pearson’s Chi-square test or Fisher’s exact test are used to analyze relationships.

A logistic regression model was used to assess the relationship between potential risk factors (adjusted by FM, VFA and BMI) and the likelihood of worst severity parameters (adjusted by VFA, BMI, HGS and PA) in patients with COVID-19.

The odds ratio (OR) was used as the effect size in these models, with a 95% confidence interval (95% CI). The significance level was set at 10%, and the software R version 4.0.3 and SPSS version 22.0 were used.

## Results

We evaluated 96 patients, with a mean age of 69.1 years (SD 15.0), divided into two groups of 48 individuals with and without COVID-19 infection. The patients that did not have COVID-19 were hospitalized because of oncological diseases (31.3%; n = 15), respiratory disorders (20.8%; n = 10), neurological diseases (18.8%; n = 9), gastrointestinal diseases (8.3%; n = 4), heart diseases (6.3%; n = 3), and genital and urinary alterations (4.2%; n = 2), other diseases (10.3%, n = 5).

**Fig. 1:**
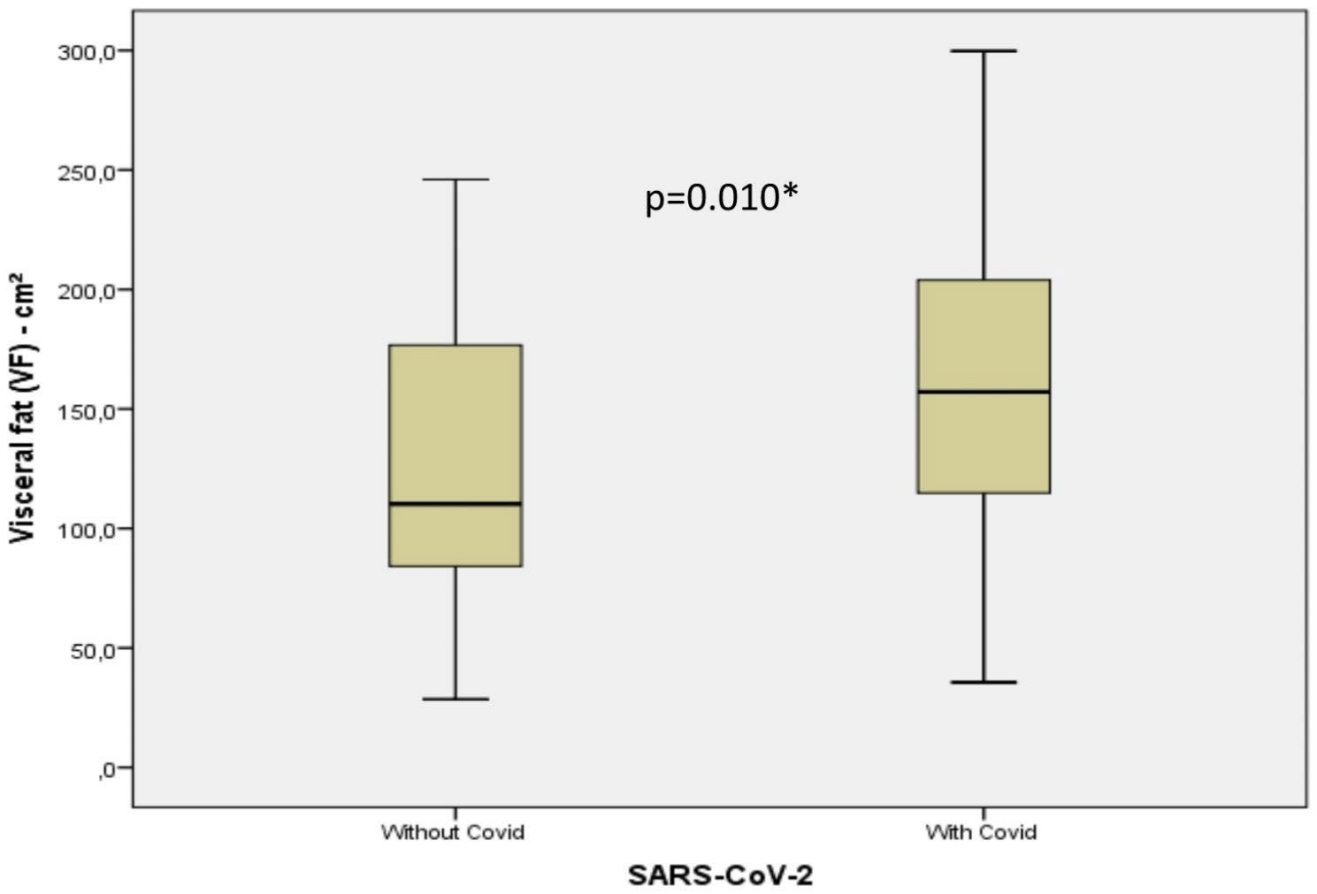
Visceral fat area measured by bioimpedance in patients with and without COVID-19 * Mann-Whitney test

**Fig. 2:**
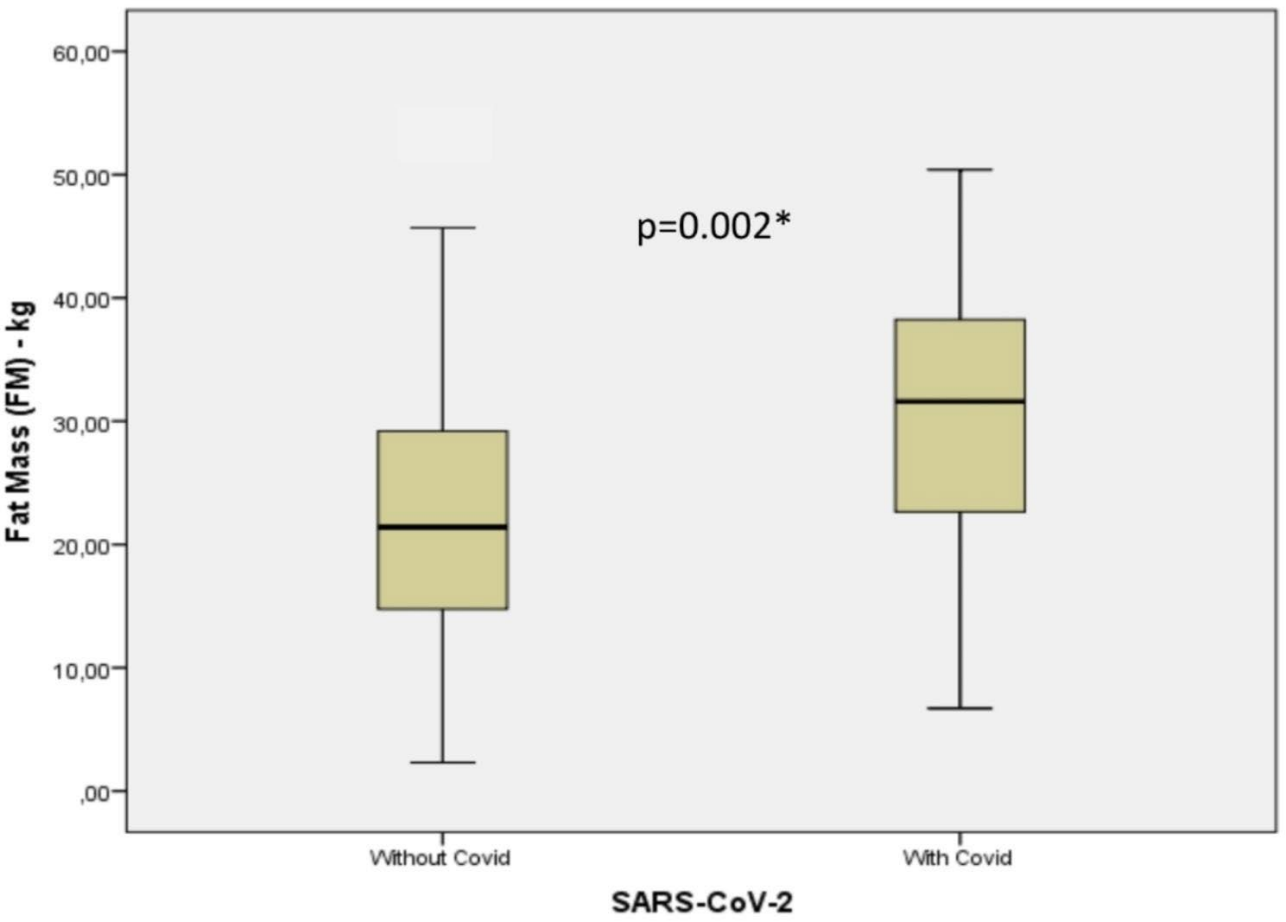
Fat mass measured by bioimpedance in patients with and without COVID-19 * Student’s t test

The characteristics of the population studied are shown in Table 1. There was a statistically significant difference between the two groups in terms of length of stay, ICU stay, need for vasoactive drugs, and OTI, as well as variables related to excess weight and body fat, indicating that BMI, FM, and VFA were higher in the presence of COVID-19 (Figs. 1 and 2). The two groups were similar concerning the other variables.

**Table 1:** Social, clinical, and nutritional characteristics of patients with and without COVID-19

Description	Without COVID-19 n = 48	With COVID-19 n = 48	p**
**Sex**			
Male	38 (79.2)	36 (75.0)	0.809
Female	10 (20.8)	12 (25.0)	
**Age group**			
Adults	8 (16.7)	16 (33.3)	0.098
Older adults	40 (83.3)	32 (66.7)	
**Comorbidities**			
Hypertension	30 (62.5)	28 (58.3)	0.835
Diabetes	14 (29.2)	18 (37.5)	0.516
Dyslipidemia	15 (31.3)	19 (39.6)	0.522
Heart diseases	18 (38.3)	11 (22.9)	0.123
Lung diseases	6 (12.5)	5 (10.4)	1.000*
**Clinical variables**			
Prolonged hospitalization (> 30 days)	22 (45.8)	32 (66.7)	0.063
Prolonged ICU stay (> 15 days)	5 (10.4)	32 (66.7)	0.000
Need for orotracheal intubation	9 (18.8)	41 (85.4)	0.000
Vasoactive drug use	14 (29.2)	34 (70.8)	0.000
**Nutritional variables**			
BMI			
Low weight	9 (18.8)	3 (6.3)	0.000
Normal weight	27 (56.3)	14 (29.2)	
Overweight	12 (25.0)	31 (64.6)	
Reduced muscle mass	10 (20.8)	7 (14.6)	0.594
High fat mass	35 (72.9)	44 (91.7)	0.052
High visceral fat area	29 (60.4)	39 (81.3)	0.042

Person Chi-Square *Fisher’s Exact Test **p-value < 0.010

It is worth noting that all the analyzed nutritional data on excess weight and body fat increased the risk of contracting COVID-19 infection (shown in Table 2).

**Table 2:** Risk of COVID-19 in relation to body fat and overweight of patients

Variable	Category	OR*	CI 95%	p***	OR**	CI 95%	p***
Fat Mass	Above	4.09	1.22 13.64	0.022	2.03	0.48 8.55	0.337
	Normal	1.00					
Visceral fat area	Above	2.84	1.12 7.18	0.027	1.08	0.33 3.53	0.903
	Normal	1.00					
Body mass index	Overweight	5.47	2.27 13.21	0.000	4.47	1.69 11.83	0.003
	Normal	1.00					

*Crude OR ** Adjusted OR ***p-value < 0.010. Multivariate models were adjusted by FM, VFA and BMI

Table 3 compares body composition data to the severity parameters of the COVID-19 patients. It is worth noting that increased VFA was found in two of the three parameters investigated.

**Table 3:** Association between severity parameters and body composition in patients with COVID-19 infection

	With COVID-19	
	n = 48	p*
**Using vasoactive drugs**		
High fat mass (n, %)	30.0 (88.2)	0.677
High visceral fat area (n, %)	25.0 (73.5)	0.043*
Reduced muscle mass (n, %)	6.0 (17.6)	0.427
**Prolonged hospital stays**		
High fat mass (n, %)	28.0 (87.5)	0.694
Visceral fat area (mean, SD)	143.6 (58.3)	0.020*
Reduced muscle mass (n, %)	5.0 (15.6)	1.000
Prolonged ICU time**		
Reduced handgrip strength (n, %)	23.0 (79.3)	0.042*
Phase angle (average, SD)	3.9 (0.8)	0.021*

ICU: intensive care unit

*p-value < 0.10 ** Prolonged ICU time (> 15 days)

In COVID-19 patients, high VFA was found in all (n = 12) female patients but in only 75% (n = 27) of male patients (*p* = 0.088). Women accumulated more visceral fat (*p* = 0.027), with a mean VFA of 187.8 cm [2] (SD 36.9) compared to 145.7 cm [2] (SD 60) for men.

There were no statistically significant differences between age and elevated VFA, with the frequency of elevated VFA being 84.4% (n = 27) in the older adults and 75% (n = 12) in adults (*p* = 0.457). There were also no statistically significant differences between age range and VFA (*p* = 0.932) between adults and the older adults [mean VFA of 155.2 cm [2] (SD 64.9) and 156.7 cm [2] (SD 54.8), respectively.

**Table 4:** Odds ratio of length of stay and length of ICU in patients with COVID-19 infection

Variable	Category	OR*	CI 95%	p***	OR**	CI 95%	p***
**Prolonged hospital stays**							
Visceral fat area^1^	Above	0.99	0.98 1.00	0.038	0.99	0.97 1.01	0.183
Body mass index	Overweight	0.30	0.07 1.25	0.097	0.72	0.10 5.03	0.743
	Normal	1.00					
**Prolonged ICU time**							
Handgrip strength	Below	4.38	1.13 16.99	0.033	3.29	0.79 13.69	0.101
	Normal	1.00					
Phase angle^1^		0.40	0.18 0.91	0.028	0.47	0.19 1.17	0.106

^1^Quantitative variable *Crude OR ** Adjusted OR ***p-value < 0.010. Multivariate models were adjusted by VFA, BMI, HGS and PA

ICU: intensive care unit

## Discussion

The results show that a high BMI is significantly associated with COVID-19 infection. This increase is most likely due to excess fat, which was also associated with the disease, and fat accumulation in the abdominal region (Table 1; Figs. 1 and 2).

High BMI increased the risk of COVID-19 infection by over 4.47 times, above-normal fat mass increased the risk by over 2 times and finally, increased AGV also led to a greater risk of developing the disease. (Table 2). This supports previous research on the relationship between obesity and body fat distribution and COVID-19 infection [6]. Worse-case scenarios include increased risks of hospitalization, ICU admission, and death among patients [3, 22]. Some factors, such as chronic inflammation in obese patients, decreased immune response, and even the adipose tissue itself, which may act as a reservoir for the virus if present primarily as visceral fat, may explain this association [5, 23].

The analysis of body composition of COVID-19 patients (Table 3) showed that an elevated visceral fat area was significantly associated with severity parameters, such as greater need for vasoactive drugs and prolonged hospital stay (over 30 days), indicating that patients with high VFA had greater disease severity, which is consistent with other studies that also associated the increase in visceral fat with a worse prognosis in patients with COVID-19 [6, 7].

The study of van Bakel et al. shows results that demonstrated a significant association of high VFA with 30-day in-hospital mortality. However, when adjusted for the 4 C Mortality Score, VFA was not associated significantly with mortality. This is in line with our study that demonstrated significant positive association only between VFA and severity parameters in patients with COVID-19 [24].

However, while total fat mass is a compelling cause of COVID-19 involvement, it is not significant in terms of severity. This is likely because, despite fat being a risk factor for COVID-19, only visceral fat has an influence on the severity of the cases, while subcutaneous fat plays no role in the progression of the disease, as supported by other studies [25]. The limited expansion capacity of adipose tissue explains why elevated VFA is a strong independent predictor of COVID-19 severity. Beyond a certain point, this tissue accommodates excess calories by producing pro-inflammatory adipocytokines [5]. Visceral adipose tissue produces three times as much interleukin 6 (IL-6) as subcutaneous fat. Increased IL-6 serum levels accumulate in the portal vein of obese patients, leading to an increase in C-reactive protein, an inflammatory marker [23].

The increased production of pro-inflammatory cytokines in obese COVID-19 patients leads to a reduced capacity to respond to the infection, which, along with the organ damage caused by the high VFA, results in a worsening response to treatments and a prolonged hospital stay in these patients, which follows our results [26].

Another explanation for the obesity and COVID-19 paradox is that visceral fat inflammation is associated with increased virus protection. Adipose tissue may play a dual role in this disease, as it has been linked to angiotensin-converting enzyme 2 (ACE2), a protein that the COVID-19 virus uses to enter cells. The ACE2 enzyme is an exoenzyme that converts the hormone angiotensin II (Ang II) into the vasodilator angiotensin 1–7. (Ang 1–7). While Ang II has proliferative, pro-inflammatory, and pro-fibrotic properties, Ang 1–7 has anti-inflammatory and antioxidant properties and is frequently reduced in metabolic dysfunctions. ACE2 expression in lung tissue is significantly reduced after viral entry [25].

Given the prevalence of high visceral fat in the population and the difficulty in reducing it, this study emphasizes the importance of monitoring body composition, including fat and its distribution as a risk factor for COVID-19 complications, to reduce disease severity.

It is well known that a decrease in PA indicates a poor prognosis for COVID-19, which is associated with cellular health damage, as shown in other diseases and conditions that affect general ICU patients [27]. In this study, we found an association between reduced PA and a longer ICU stay (Table 3). In addition to each degree of reduction of AF increased the patient’s risk of having a prolonged ICU stay by 0.47 times (Table 4). Therefore, while there are no published reference values for PA in the literature to determine the increased risk of severe COVID-19 cases, monitoring PA values throughout the patient’s hospitalization could help us assess how patients are progressing and implement specific interventions for patients with reduced PA between one evaluation and another.

Using BIA instead of computed tomography (CT) to determine VFA was a strength of our study because, although CT is considered the gold standard, BIA has several advantages over CT. BIA is a non-invasive and low-cost method test without exposure to radiation. Requires less scanning time and is easy and safe to measure and therefore suitable not only for everyday use clinical practice, but also for epidemiological studies in a large scale [9]. Furthermore, it enabled us to measure PA, which is an important parameter to monitor, particularly in patients with more severe disease.

Yoon et al. showed that the mean difference between CT to BIA to determine VFA is close to zero. However, in the context of recent knowledge, application of a BIA machine combined with a portable abdominal BIA device improved the correlation with CT-measured [9].

Handgrip strength measurement is also recommended to estimate overall impairment in COVID-19 patients [28]. Our results show that patients with reduced handgrip strength were 3.29 times more likely to require a prolonged ICU stay (Table 4). This could be because increased levels of interleukin-6, which have previously been associated with muscle atrophy and decline in COVID-19 patients, are associated with atrophy and reduced muscle function. Another hypothesis is that patients with COVID-19 have increased endothelial cell expression, which is related to the coagulopathy present in these patients causing hypoxia, which can negatively impact the patients’ muscle strength [28].

Furthermore, handgrip strength reflects the patient’s overall strength, including respiratory function. Therefore, when reduced, it can have a significant impact on the patient’s response to the disease and thus the length of hospital stays, which is consistent with our results [29]. Accordingly, we believe that handgrip strength can be used to assess the severity of COVID-19 patients.

There was no association between reduced muscle mass and the presence of COVID-19, which could be attributed to the population of the present study coming from a private hospital with better social conditions, which contributes to a better nutritional status at hospital admission.

Some limitations should be considered when interpreting the results: the study population is heterogeneous but reflects the population treated in private hospitals, BIA was not performed on fasting patients; and diuretics were not stopped. However, these behaviors follow hospital reality, where medications cannot be stopped, and patients cannot always fast for extended periods of time.

## Conclusion

Our results showed that excess weight and body fat are important for COVID-19 involvement, but the severity is primarily related to a largest visceral fat area. In this context, using bioimpedance for visceral fat measurement proved effective, being a simple method to use in a hospital setting and one that does not require the use of radiation. In addition, bioimpedance can determine the PA of these patients, which may be a marker for disease severity. We also showed that muscle strength, when reduced, has a negative impact on patient outcome.

## Data Availability

The data used and/or analyzed during the current study are available with the corresponding author upon request.

## List of abbreviations

ACE2: Angiotensin-converting enzyme 2
Ang 1: 7-Aasodilator angiotensin 1-7
Ang II: Hormone angiotensin II
BIA: Bioimpedance electrical
BMI: Body mass index
FM: Fat Mass
HGS: Handgrip strength (HGS)
ICU: Intensive care unit
OR: Odds ratio
OTI: Orotracheal intubation
PA: Phase angle
REC: Research Ethics Committee
VFA: Visceral fat area
WHO: World Health Organization
